# Evaluation of a large set of patients with Autoimmune Polyglandular Syndrome from a single reference centre in context of different classifications

**DOI:** 10.1007/s40618-023-02200-6

**Published:** 2023-09-26

**Authors:** E. Gatta, V. Maltese, E. Cimino, M. Cavadini, V. Anelli, E. Di Lodovico, E. Piovani, I. Zammarchi, G. Gozzoli, B. Agosti, I. Pirola, A. Delbarba, A. Girelli, C. Buoso, F. Bambini, D. Alfieri, W. Bremi, P. Facondo, R. Lupo, F. Bezzi, M. Fredi, A. M. Mazzola, E. Gandossi, M. Saullo, F. Marini, M. Licini, L. C. Pezzaioli, L. Pini, F. Franceschini, C. Ricci, C. Cappelli

**Affiliations:** 1 Department of Clinical and Experimental Sciences, SSD Endocrinologia, University of Brescia, ASST Spedali Civili of Brescia, Piazzale Spedali Civili no 1, 25100 Brescia, Italy; 2 UOC Medicina Generale ad Indirizzo Metabolico e Diabetologico, ASST Spedali Civili of Brescia, Brescia, Italy; 3 Sindacato Unico Medicina Ambulatoriale Italiana e Professionalità dell’Area Sanitaria—SUMAI, Trade Union Organisation, Brescia, Italy; 4 Department of Clinical and Experimental Sciences, Rheumatology and Clinical Immunology, University of Brescia, ASST Spedali Civili of Brescia, Brescia, Italy; 5 Department of Clinical and Experimental Sciences, Gastroenterology Unit, University of Brescia, ASST Spedali Civili of Brescia, Brescia, Italy; 6 Department of Clinical and Experimental Sciences, Respiratory Medicine Unit, University of Brescia, ASST Spedali Civili of Brescia, Brescia, Italy

**Keywords:** Autoimmune polyglandular syndrome, Autoimmune diseases, Autoimmunity

## Abstract

**Purpose:**

To characterize patients with APS and to propose a new approach for their follow-up. Query ID="Q1" Text="Please check the given names and familynames."

**Methods:**

Monocentric observational retrospective study enrolling patients referred to the Outpatients clinic of the Units of Endocrinology, Diabetology, Gastroenterology, Rheumatology and Clinical Immunology of our Hospital for Autoimmune diseases.

**Results:**

Among 9852 patients, 1174 (11.9%) [869 (73.9%) female] were diagnosed with APS. In 254 subjects, the diagnosis was made at first clinical evaluation (Group 1), all the other patients were diagnosed with a mean latency of 11.3 ± 10.6 years (Group 2). Group 1 and 2 were comparable for age at diagnosis (35.7 ± 16.3 vs. 40.4 ± 16.6 yrs, p = .698), but different in male/female ratio (81/173 vs 226/696, p = .019). In Group 2, 50% of patients developed the syndrome within 8 years of follow-up. A significant difference was found after subdividing the first clinical manifestation into the different outpatient clinic to which they referred (8.7 ± 8.0 vs. 13.4 ± 11.6 vs. 19.8 ± 8.7 vs. 7.4 ± 8.1 for endocrine, diabetic, rheumatologic, and gastroenterological diseases, respectively, p < .001).

**Conclusions:**

We described a large series of patients affected by APS according to splitters and lumpers. We propose a flowchart tailored for each specialist outpatient clinic taking care of the patients. Finally, we recommend regular reproductive system assessment due to the non-negligible risk of developing premature ovarian failure.

**Supplementary Information:**

The online version contains supplementary material available at 10.1007/s40618-023-02200-6.

## Introduction

Autoimmune polyendocrine (or polyglandular) syndromes (APS) are rare orphan diseases (ORPHAcode ORPHA:282196) encompassing a wide spectrum of autoimmune diseases, with the involvement of endocrine and non-endocrine organs [1, 2]. APS display a great heterogeneity of syndromes and manifest sequentially with variable intervals between the onset of the diseases [3]. The original classification in four types by Neufeld et al*.* in 1980 was revised by Betterle and Zanchetta in 2003, sub-classifying APS type 3 in four different sub-groups [4, 5].

APS-1 (ORPHA:3453), also known as APECED (Autoimmune polyendocrinopathy candidiasis ectodermal dystrophy), is typically a childhood syndrome, with an estimated prevalence of 1–9:1,000,000 live births [1]. Chronic mucocutaneous candidiasis, chronic hypoparathyroidism and Addison's disease are the characteristic disorders of this syndrome [6, 7]. Inheritance is monogenic, autosomal recessive, and it is determined by mutations in the AIRE (AutoImmune REgulator) gene leading to the lack of apoptosis of lymphocytes directed towards self-proteins.

Adult APS types are polygenic syndromes. Genetic predisposition is determined by class II human leucocyte antigen (HLA) genes on chromosome 6 and polymorphisms of non-HLA genes involved in autoimmune T-mediated lymphocyte responses [8].

APS-2 (ORPHA:3143) has an estimated prevalence of 1:20,000 and sex ratio male/female 1:3 and is characterized by the presence of Addison’s disease associated with autoimmune thyroid diseases and/or type 1 diabetes [8]. Other nonendocrine autoimmune disorders, such as vitiligo, myasthenia gravis, thrombocytopenic purpura, Sjogren’s syndrome, rheumatoid arthritis, and primary antiphospholipid syndrome, occur occasionally [3, 9, 10].

APS-3 (ORPHA:227982) includes autoimmune thyroid diseases plus another autoimmune disorder in the absence of Addison’s disease and/or hypoparathyroidism. This syndrome presents an estimate incidence of 1:20,000 live births, and with a male/female ratio of 1:3 [11, 12]. Different HLA class II alleles correlated with APS-3 but with other associated genes include those encoding lymphoid tyrosine phosphatase (PTPN22) and cytotoxic T Lymphocyte associated antigen-4 (CTLA-4) [13, 14].

APS-4 (ORPHA:227990) includes all the different clinical combinations of autoimmune diseases not included in the previous groups and affecting an endocrine organ (with the exception of Addison’s disease, thyroid diseases, or hypoparathyroidism) in combination with at least one more endocrine or non-endocrine organs [1, 2, 15]. Its prevalence hasn’t been reported yet.

However, few Authors proposed an alternative classification including only two major subtypes of APS: APS-1, and APS-2. The last one, should be a more common polygenic variety including APS-3 and APS-4 (2, 10).

The clinical presentation of autoimmune polyendocrine syndromes is often preceded by a long asymptomatic phase characterized by the isolated presence of circulating antibodies. The early detection of these antibodies facilitates the diagnosis of autoimmune endocrine disorders, allowing early damage and secretory loss of function to be prevented. In addition, the possibility of a standardized follow-up for each APS type and for different autoimmune diseases that are potential “drivers” for developing APS could enable a personalized clinical evaluation of each patient, regardless the classification adopted. The aim of the present study was firstly to characterize epidemiologically and clinically all the APS diagnosed and followed in our Reference Centre; and secondly, to propose a new approach for their follow-up.

## Methods

### Patients

We reviewed retrospectively the medical records of all patients with autoimmune diseases diagnosed and followed at outpatient clinics for Endocrinology, Diabetology, Gastroenterology, Rheumatology and Clinical Immunology at our Reference Centre for APS, from January 1, 2000, to March 31, 2023. All patients affected by Autoimmune Polyglandular Syndrome were included in this study.

### Clinical data collection

The clinical records reviewed included all patients affected by type 1 diabetes mellitus, autoimmune thyroiditis, Graves’ disease, Addison’s disease, chronic hypoparathyroidism, premature ovarian failure, celiac disease, chronic atrophic gastritis, inflammatory bowel disease, rheumatoid arthritis, systemic lupus erythematosus, scleroderma, Sjogren syndrome, mixed connective tissue disease, vasculitis, antiphospholipid syndrome, primary biliary cirrhosis, autoimmune hepatitis, alopecia areata, autoimmune urticaria, myasthenia gravis, multiple sclerosis, pernicious anemia, immune thrombocytopenia, vitiligo, seronegative arthritis, ankylosing spondylitis, psoriasis, pemphigoid, and chronic mucocutaneous candidiasis.

Demographic and anthropometric data, date of diagnosis, family history for autoimmune diseases, serological and/or histological data, and need for medical therapy were collected anonymously in an electronic database.

The study (ASST_BS_CLIN_PZ_SPA-BS) was approved by the local Ethics Committee (no 5517).

### Statistical analysis

Normal distribution was achieved using the Shapiro–Wilk test. Between-group comparison was performed using the Student’s T-test for unpaired data or ANOVA for quantitative variables, as appropriate, and the χ^2^ test for categorical variables. Kruskal–Wallis test was used to compare median latency. The Kaplan–Meier curve was fitted to determine the APS diagnosis time, and boxplot to represent latency. A p-value < 0.05 was considered statistically significant. The statistical analyses were performed using SPSS 20.0 software (SPSS, Inc., Evanston, IL, USA). The results are reported in compliance with the STROBE reporting guidelines for cross-sectional studies; the checklist is reported in Supplementary file 1.

## Results

Among the 9852 individuals referred to outpatient clinics for Endocrinology, Diabetology, Gastroenterology, Rheumatology and Clinical Immunology at our institute and included in the study for a driver disease, 1174 (11.9%) [869 (73.9%) female] were diagnosed with an Autoimmune Polyglandular Syndrome in 99,342 person-years follow-up.

In 254 subjects, the diagnosis was made at first clinical evaluation for the simultaneous presence of at least two diseases characterizing APS (Group 1). All the other patients were diagnosed during follow-up with a mean latency of 11.3 ± 10.6 years (Group 2). The two groups were comparable for age at diagnosis (35.7 ± 16.3 vs. 40.4 ± 16.6 yrs, p = 0.698), but different in male/female ratio (81/173 vs 226/696, p = 0.019).

**Fig. 1:**
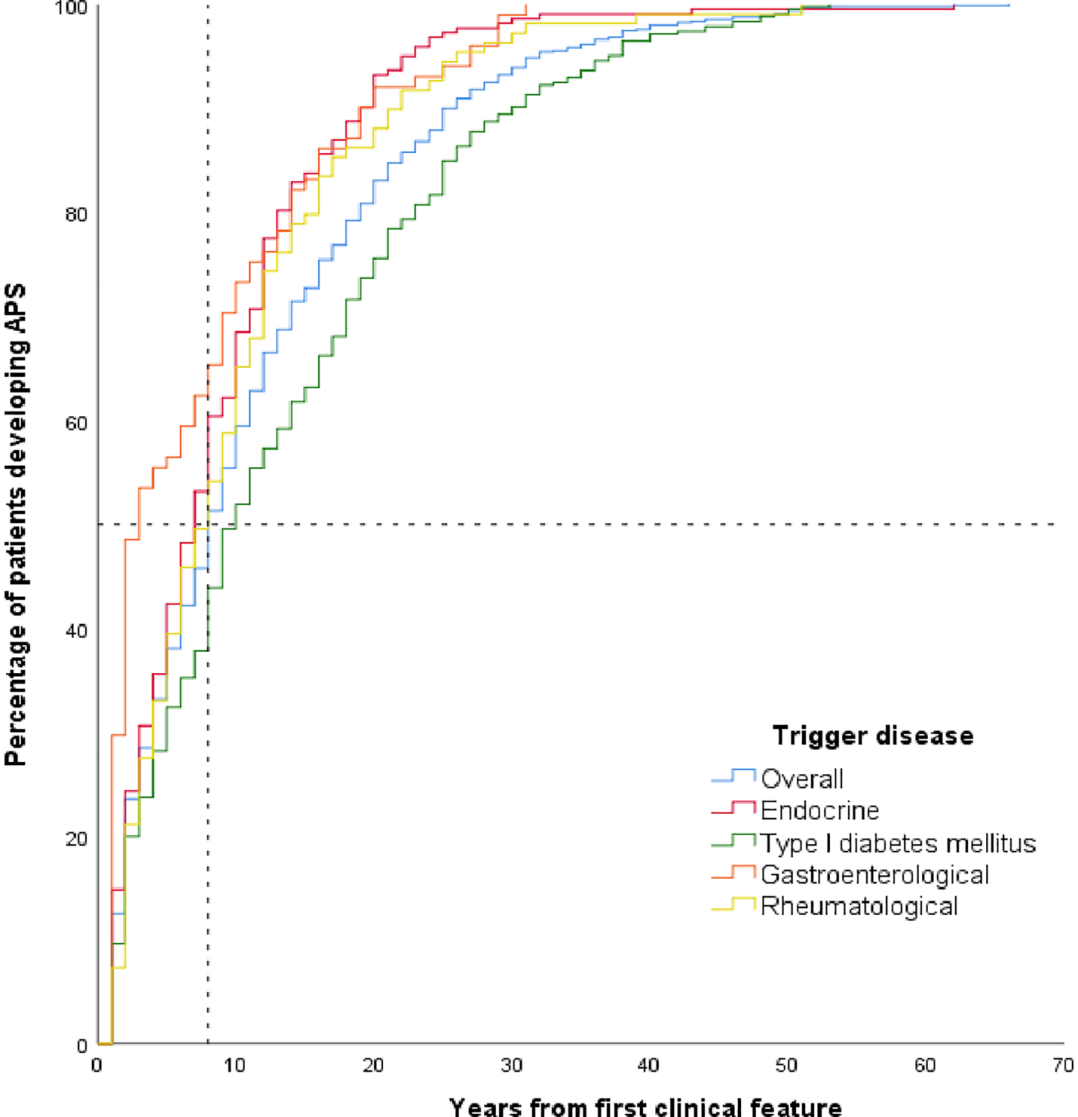
Timeline of APS development in accordance with the different outpatient clinic to which patients referred

In Group 2, 50% of patients developed the syndrome within 8 years of follow-up. A significant difference was found after subdividing the first clinical manifestation into the different outpatient clinic to which they referred (8.7 ± 8.0 vs. 13.4 ± 11.6 vs. 19.8 ± 8.7 vs. 7.4 ± 8.1 for endocrine, diabetic, rheumatologic, and gastroenterological diseases, respectively, p < 0.001) (Fig. 1).

### Characteristics of each type of APS according to original classification—the splitters

In detail, among 1174 patients, 2 (1/1, M/F) were affected by APS1, 16 (7/9) by APS-2, 1046 (248/798) by APS-3 and 111 patients (51/61) by APS-4 (Table 1). At the time of diagnosis, they were 40.4 ± 16.6 years old, with no differences in mean age between sex (39.0 ± 17.6 vs. 40.8 ± 16.3 yrs, M/F, p = 0.120).

**Table 1:** Demographic characteristics of the patients

	All (*n* = 1174)	APS-1 (*n* = 2)	The splitters				The lumpers
			APS-2 (*n* = 16)	APS-3 (*n* = 1045)	APS-4 (*n* = 111)	*p*	APS-2 (*n* = 1172)
Male/female	307/869	1/1	7/9	248/797	51/60	.000	306/866
Mean age at diagnosis (years)	40.4 ± 16.6	**–**	43.9 ± 12.7	39.9 ± 16.6	34.5 ± 16.8	.000	40.4 ± 16.6
Male	39.0 ± 17.6	**–**	46.4 ± 14.0	40.5 ± 16.4	34.1 ± 17.6	.068	39.0 ± 17.6
Female	40.8 ± 16.3	**–**	42.0 ± 12.0	37.9 ± 17.1	34.9 ± 16.2	.005	40.8 ± 16.3
Diagnosis at the onset (n, %)	253 (21.6%)	2 (100%)	5 (31.3%)	233 (22.3%)	15 (13.5%)	.006	253 (21.6%)
Latency before diagnosis (years)	10.9 ± 10.3	**–**	9.5 ± 8.7	11.0 ± 10.6	13.5 ± 10.3	.083	10.9 ± 10.3
Male	12.1 ± 11.5	**–**	12.0 ± 7.6	11.7 ± 11.5	14.0 ± 11.7	.519	12.1 ± 11.5
Female	11.0 ± 10.2	**–**	8.5 ± 9.4	10.8 ± 10.3	13.1 ± 9.2	.237	11.0 ± 10.2

#### APS-1 (ORPHA:3453)

Two (0.2%) subjects were affected by APS-1: a 29-year-old man with chronic mucocutaneous candidiasis and hypoparathyroidism diagnosed at 7 years old, and an asymptomatic 40-year-old woman. The patient, who had a strong desire for having children, underwent genetic testing after a premature death of her daughter affected by an unspecified genetic syndrome.

#### APS-2 (ORPHA:3143)

APS-2 was diagnosed in 16 (1.4%) patients [9 (56.3%) females], with a mean age at diagnosis of 43.9 ± 12.7 years, comparable between sex (46.4 ± 14.0 vs. 42.0 ± 12.0 yrs, M/F, p = 0.726). Five (31.3%) subjects (four male) showed APS-2 at the first clinical evaluation by the concomitant presence of Addison’s disease and autoimmune thyroiditis and the woman also had premature ovarian failure (POF); none developed further autoimmune diseases during follow-up (18 person-years). Eleven (68.7%) patients (eight female) developed APS after a mean latency of 9.5 ± 8.7 years. Autoimmune thyroids were the first clinical feature in five patients and Addison’s disease in one (Supplementary file 2). Addison’s disease developed with a mean latency of 10.3 ± 8.7 from the first clinical feature.

#### APS-3 (ORPHA:227982)

APS-3 was diagnosed in 1045 (89.1%) patients, [797 (76.3%) females] with a mean age of 39.9 ± 16.6 years, comparable between sex (40.5 ± 16.4 vs. 37.9 ± 17.1 yrs, M/F, p = 0.413). The syndrome was diagnosed at first evaluation in 232 (22.2%) subjects (68 males) with the concomitant presence of two diseases characterizing APS-3 (Group 1). Among them, 33 (14.2%) patients (seven males) developed other autoimmune diseases (Supplementary file 3). No differences in sex (7/26 vs. 61/138, M/F, p = 0.270) and age (36.8 ± 17.0 vs. 36.8 ± 16.0, p = 0.471) were found between patients that developed or did not develop subsequent diseases during the 2352 person-years of follow-up. In 813 patients [633 (77.9%) women], the diagnosis was made after a mean latency of 11.0 ± 10.6 from the onset of the first “driver” manifestation (Group 2). The age of diagnosis was comparable between Groups 1 and 2 (36.7 ± 16.1 vs. 40.8 ± 16.6 yrs, p = 0.167) but the gender distribution was different (70% vs 78% women, p = 0.018). Epidemiological and clinical data are reported in Supplementary file 3. In detail, autoimmune thyroid disease was most frequently associated with type 1 diabetes mellitus in 502 (48.0%) patients, celiac disease in 237 (22.7%), and rheumatoid arthritis in 152 (14.5%).

#### APS-4 (ORPHA:227990)

APS-4 was diagnosed in 111 (9.5%) patients, [60 (54.1%) women] with mean age of 34.5 ± 16.8 years, comparable between sex (34.1 ± 17.6 vs. 34.9 ± 16.2 yrs, M/F, p = 0.195). The diagnosis was made during the first clinical evaluation in 15 (13.5%) patients (8 men) with the concomitant presence of two diseases characterizing the syndrome (Group 1). None developed further autoimmune diseases during the 215 person-years of follow-up. In the other 96 patients [53 (55.2%) women] the diagnosis was made after a mean latency of 13.5 ± 10.3 years from the onset of the first “driver” manifestation (Group 2). Age at diagnosis and sex were comparable for Groups 1 and 2 (23.5 ± 13.3 vs. 23.0 ± 15.4 yrs, p = 0.292; 47% vs 55% women, p = 0.537). The epidemiological and clinical data are reported in Supplementary file 4. In detail, type 1 diabetes mellitus was most frequently associated with Celiac disease in 55 (49.5%) patients, vitiligo 10 (9%), and rheumatoid arthritis in 10 (9%).

### Characteristics of each type of APS according to alternative classification—the lumpers

APS-1 (ORPHA:3453) was already above described.

APS-2 (polygenic variety) was diagnosed in 1172 (99.8%) patients, [866 (73.9%) women] with mean age of 40.4 ± 16.6 years, comparable between sex (39.0 ± 17.6 vs. 40.8 ± 16.3 yrs, M/F, p = 0.120). The diagnosis was made during the first clinical evaluation in 253 (21.6%) patients (77 men) for the concomitant presence of two diseases characterizing the syndrome (Group 1); 34 patients developed further autoimmune diseases. In the other 919 patients [694 (75.5%) women] the diagnosis was made after a mean latency of 10.9 ± 10.3 years from the onset of the first “driver” manifestation (Group 2). Groups 1 and 2 differed for age at diagnosis and sex (36.0 ± 16.1 vs. 39.0 ± 17.6 yrs, p = 0.024; 70% vs 76% women, p = 0.006).

## Discussion

Autoimmune polyglandular syndrome encompasses a variety of rare diseases that are, however, coming increasingly under the radar among practitioners. This is well evidenced by the significant increase of published data, particularly in the last 10 years. This syndrome is recognized in four different types according to the original Orphanet classification [1]. However, an alternative classification was recently proposed considering APS-1, and APS-2, including APS-3 and APS-4 [2, 8]. In our survey, Autoimmune polyglandular syndromes were diagnosed in 1174/9852 subjects (11.9%) referred to our outpatient clinic (Endocrinology, Diabetology, Gastroenterology, Rheumatology and Clinical Immunology) for at least one autoimmune disease. These data, in agreement with the original classification, adjusted to the residents (1,253,993) of our province [16], should estimate a prevalence of APS-1, APS-2, and APS-4 of 2:1,000,000, 1:100,000, and 9:100,000 live births, as expected by literature data [1, 8]. However, the estimated prevalence of APS-3 is 8:10,000, 16-fold higher than expected (1:20,000) [11, 12], which means the syndrome can no longer be classified as a rare disease [1]. Frommer and Kahaly indeed hypothesized a higher number of unreported cases owing to the heterogeneous expression pattern of this syndrome [11]. In agreement, Betterle et al*.* estimated that 1/3 of women and 1/4 of men suffering from autoimmune thyroid diseases (AITDs) will develop APS-3 [17]. Moreover, we must underline that AITDs are largely diagnosed in Western countries [14]; in particular, early post-mortem studies confirmed histological evidence of chronic autoimmune thyroiditis in 27% of adult women and 7% of men [18]. These data seem to support our findings and in fact it could be assumed that APS-3 cases reported worldwide represent only the tip of the iceberg. However, according to the alternative classification, the estimate prevalence of APS-2 is 9:10.000, 18-fold higher than reported [19].

Overall, regardless of the classification of APS adopted, one-fifth of our patients was diagnosed at first evaluation with the concomitant presence of at least two diseases; the others developed APS with a mean latency of 11.3 years. The two groups were comparable for age at diagnosis but, in terms of sex, APS was more frequently diagnosed at first evaluation in men than in women (p = 0.019). We have no explanation for these data, we can only hypothesize the different role of sexual hormones in inflammatory response. It is well known that the male sex has a protective role in the development of autoimmune disease, and it is likely that this is due to sexual hormones and the Y chromosome [20, 21]. Several studies indicate that testosterone has suppressive effects on the immune system by inhibiting pro-inflammatory cytokine and immunoglobulin production, T-helper 1 (Th1) differentiation, natural killer (NK) cell cytotoxic activity and by potentiating the expression of anti-inflammatory cytokines [20]. By contrast, female-predominant autoimmune diseases have a clear antibody-mediated pathology characterized by long-standing chronic inflammation leading to fibrosis [22]. The flipside of the coin was evidenced by Fairweather et al*.* showing that male-predominant autoimmune diseases are characterized by acute Th-1 mediated inflammation [22]. Thus, we can hypothesize that in male subjects, when the protection system fails, the autoimmunity cascade occurs early and is more aggressive, explaining, at least in part, our findings.

Among patients with a “driver” disease, 9.6% developed APS with a mean latency of 11.3 years, up to 46 years. The heterogeneity and the complexity of high number of possible combinations of autoimmune diseases make it difficult or even impossible to create a recommended “universal” flowchart for the screening of each “driver” disease to be used in the routine clinical practice. Van den Driessche et al*.* suggested timeline checkups for patients affected by T1DM. The authors proposed an annual screening for the first 3 years and then once every 5 years for celiac and Addison’s disease, atrophic gastritis and vitiligo, whereas the thyroid should be checked annually [23]. For patients affected by Addison’s disease, Kahaly and Frommer suggested screening for autoimmune thyroid disease and Type 1 diabetes mellitus every few years [19]. The Guidelines of the European Society of Human Reproduction and Embryology (ESHRE) recommends routine screening for autoimmune thyroid disease and Addison’s disease for patients affected by premature ovarian failure [24]. In addition, Weetman recommended screening for chronic atrophic gastritis, Addison’s disease, lymphocytic hypophysitis, isolated ACTH deficiency, primary biliary cirrhosis, celiac disease, and myasthenia gravis, at first evaluation, for patients affected by autoimmune thyroid diseases, whereas routine testing for autoantibodies associated with rheumatological disorders is not recommended in the absence of signs or symptoms [25].

**Fig. 2:**
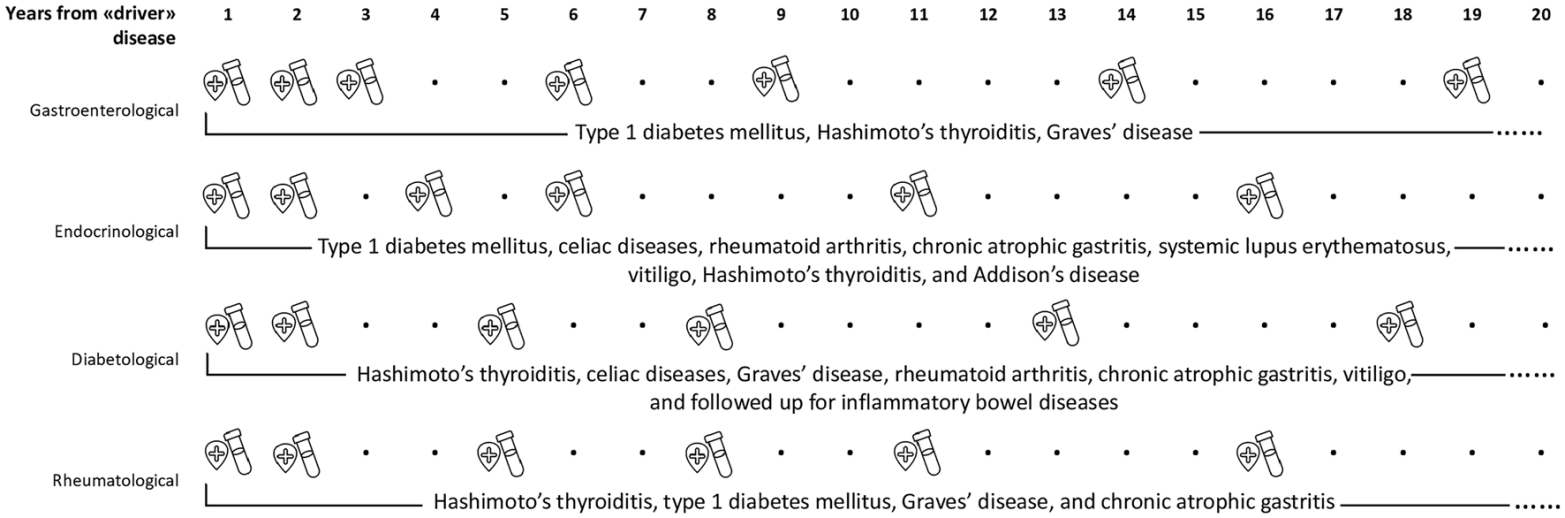
Follow-up flowchart based on the outpatient clinic to which patients referred to. The crossed shield denotes clinical evaluation while the tube blood test

Differently, we propose an arbitrary flowchart tailored for each outpatient clinic (Endocrinology, Diabetology, Gastroenterology, Rheumatology and Clinical Immunology) with the aim to simplify the follow-up of this complexity of autoimmune associations in routine clinical practice. In other words, we shifted the focus from the “driver” to the different clinical specializations. We demonstrated that patients diagnosed with a gastroenterological disease developed APS much earlier than the others (p = 0.001), suggesting the need for closer follow-up in the early years (Fig. 1), as 50% of them developed APS within 3 years of follow-up. In line with this new perspective, we propose a “outpatient timeline” (Fig. 2**)**. We suggest a complete screening at the onset of each “driver” disease including antibodies for the most frequently associated disorder. Subsequently, patients with gastroenterological impairments should be screened annually for the first 3 years and then every 3 years over the following 6 years only with functional organ testing. The most frequently associated disorders that should be looked for are type 1 diabetes mellitus, Hashimoto’s thyroiditis and Graves’ disease. Endocrinological outpatients should be checked annually for the first 2 years and then examined every 2 years for the following 6 years for type 1 diabetes mellitus, celiac diseases, rheumatoid arthritis, chronic atrophic gastritis, systemic lupus erythematosus, vitiligo, Hashimoto’s thyroiditis and Addison’s disease. Patients affected by diabetes mellitus should be checked annually for the first 2 years and then every 2 years for Hashimoto’s thyroiditis, celiac diseases, Graves’ disease, rheumatoid arthritis, chronic atrophic gastritis, vitiligo, with follow-up also for inflammatory bowel diseases**.** Rheumatological patients should be checked annually for the first 2 years and then every three years for Hashimoto’s thyroiditis, type 1 diabetes mellitus, Graves’ disease and chronic atrophic gastritis. After this tailored follow up, a lifelong follow-up with screening every 5 years is suggested for all patients.

Finally, particular attention must be paid to the possible development of premature ovarian failure, a condition that severely affects women’s lives [26–29]. For this reason, it is important to emphasize that 13 (2.2%) of women in our series developed POF. Our data showed a lower incidence than previously reported [30, 31] considering the splitters, but with different prevalence among the lumpers (14.3% of women affected by APS-2, 1.7% by APS-3 and 5.7% by APS-4). Anti-Mullerian hormone (AMH) is considered a reliable tool for recognition and risk assessment of developing POF in patients with other autoimmune diseases [32]. In particular, Saglam et al*.* showed that women with autoimmune thyroid disease appear to have a diminished ovarian follicular reserve and measurement of the serum AMH level can be used to predict this comorbidity [33]. For this reason, we recommend that gynecologists perform regular check-ups for these patients with complete blood exams during childbearing age.

The main limitations of the present study are its retrospective nature and the possible patient drop-out during follow-up after the diagnosis of the first “driver” disease. The latter is a key point, as it could reduce the prevalence of APS in our cohort of patients. Unfortunately, we have no data on patient drop-out precisely due to the retrospective nature of the study. However, the large set of patients, the careful selection procedure and detailed analysis of patient clinical records strengthen our results. Finally, only patients with clinical presentation of the syndromes were enrolled in the study; by the fact, it is possible that earlier stages of autoimmune diseases (mainly subclinical) were not included.

In conclusion, we described a large series of patients affected by APS in accordance to splitters and lumpers. Different flowcharts for patient screening and follow-up have been proposed based on the type of APS or the first “driver” disease diagnosed. We instead propose a flowchart tailored for each specialist outpatient clinic taking care of the patients. Finally, we recommend regular reproductive system assessment with complete blood tests during childbearing age due to the non-negligible risk of developing premature ovarian failure. Even though these recommendations stem from an extensively collected data and a long follow-up period, they appear arbitrary also due to the complexity of autoimmune diseases associations. For this reason, prospective large studies are needed to confirm this intriguing point of view.

### Supplementary Information

Below is the link to the electronic supplementary material.

Supplementary file1 (DOC 91 KB)

Supplementary file2 (DOCX 15 KB)

Supplementary file3 (DOCX 39 KB)

Supplementary file4 (DOCX 18 KB)

## Data Availability

Original data generated and analyzed during this study are included in this published article.
