# Supplementary material for: Evaluation of a large set of patients with Autoimmune Polyglandular Syndrome from a single reference centre in context of different classifications
Source: J Endocrinol Invest. 2023 Sep 26;47(4):857–64. doi: 10.1007/s40618-023-02200-6 (PMC10965644; doi:10.1007/s40618-023-02200-6)
Supplement: Supplementary file 2 — Supplementary file2 (DOCX 15 KB) [file 40618_2023_2200_MOESM2_ESM.docx]

**Supplementary** **file 2.** Demographic and clinical characteristics of patients diagnosed with APS-2.

| **First disease** | **Number of patients (%)** | **Sex M/F** | **Mean age of diagnosis** | **Latency from APS diagnosis and range (years)** | **Subsequent diseases** | **Number of patients (%)** | **Latency from the first disease and range (years)** |
| --- | --- | --- | --- | --- | --- | --- | --- |
| Autoimmune thyroiditis | 5 (33.3%) | 1/4 | 39.8 ± 14.0 | 7.6 ± 7.7 (1-20) | Addison’s disease | 5 (100%) | 8.2 ± 7.3 (2-20) |
|  |  |  |  |  | Chronic atrophic gastritis | 2 (40%) | 7.0 ± 4.2 (4-10) |
|  |  |  |  |  | Sjogren syndrome | 1 (20%) | 3 |
|  |  |  |  |  | Primary biliary cirrhosis | 1 (20%) | 1 |
| Vitiligo | 3 (20%) | 2/1 | 33.3 ± 15.3 | 13.7 ± 7.8 (5-20) | Addison’s disease | 2 (66%) | 22.0 ± 8.5 (5-28) |
|  |  |  |  |  | Autoimmune thyroiditis | 1 (33%) | 20 |
|  |  |  |  |  | Chronic atrophic gastritis | 1 (33%) | 41 |
|  |  |  |  |  | Addison’s disease and Graves’ disease | 1 (33%) | 5 |
| Addison’s disease and autoimmune thyroiditis | 3 (20%) | 3/0 | 42.0 ± 13.0 | 0 | NA | NA | NA |
| Addison’s disease | 1 (6.7%) | 0/1 | 38 | 1 | Autoimmune thyroiditis | 1 (100%) | 5 |
| Graves’ disease | 1 (6.7%) | 1/0 | 51 | 11 | NA | NA | NA |
| Type I diabetes mellitus | 1 (6.7%) | 0/1 | 36 | 2 | Addison's disease and autoimmune thyroiditis | 1 (100%) | 2 |
| Addison’s disease, autoimmune thyroiditis and premature ovarian failure | 1 (6.7%) | 0/1 | 33 | 0 | NA | NA | NA |

NA: not applicable
