# Supplementary material for: Evaluation of a large set of patients with Autoimmune Polyglandular Syndrome from a single reference centre in context of different classifications
Source: J Endocrinol Invest. 2023 Sep 26;47(4):857–64. doi: 10.1007/s40618-023-02200-6 (PMC10965644; doi:10.1007/s40618-023-02200-6)
Supplement: Supplementary file 3 — Supplementary file3 (DOCX 39 KB) [file 40618_2023_2200_MOESM3_ESM.docx]

**Supplementary** **file 3.** Demographic and clinical characteristics of patients diagnosed with APS-3.

| **First disease(s)** | **Number of patients (%)** | **Sex M/F** | **Mean age of diagnosis** | **Latency from APS diagnosis and range (years)** | **Subsequent diseases** | **Number of patients (%)** | **Latency from the first disease and range (years)** |
| --- | --- | --- | --- | --- | --- | --- | --- |
| Type I diabetes mellitus | 355 (33.9%) | 118/237 | 22.7 ± 15.2 | 13.0 ± 1.5 (1-53) | Autoimmune thyroiditis | 336 (94.6%) | 13.5 ± 11.9 (1-53) |
|  |  |  |  |  | Celiac disease | 24 (6.8%) | 12.7 ± 11.9 (1-40) |
|  |  |  |  |  | Graves’ disease | 19 (5.4%) | 11.8 ± 8.5 (1-30) |
|  |  |  |  |  | Chronic atrophic gastritis | 10 (2.8%) | 21.6 ± 17.3 (3-56) |
|  |  |  |  |  | Rheumatoid arthritis | 5 (1.4%) | 21.6 ± 15.2 (4-43) |
|  |  |  |  |  | Inflammatory bowel disease | 2 (0.6%) | 17.0 ± 11.3 (9-25) |
|  |  |  |  |  | Vitiligo | 2 (0.6%) | 24.5 ± 20.5 (10-39) |
|  |  |  |  |  | Scleroderma | 1 (0.3%) | 24 |
|  |  |  |  |  | Sjogren’s syndrome | 1 (0.3%) | 12 |
|  |  |  |  |  | Antiphospholipid syndrome | 1 (0.3%) | 9 |
|  |  |  |  |  | Vasculitis | 1 (0.3%) | 26 |
|  |  |  |  |  | Primary biliary cirrhosis | 1 (0.3%) | 22 |
|  |  |  |  |  | Autoimmune urticaria | 1 (0.3%) | 2 |
|  |  |  |  |  | Systemic lupus erythematosus | 1 (0.3%) | 8 |
|  |  |  |  |  | Seronegative arthritis | 1 (0.3%) | 18 |
| Autoimmune thyroiditis | 174 (16.7%) | 157/17 | 44.5 ± 15.4 | 7.9 ± 6.4 (1-32) | Type I diabetes mellitus | 58 (33.3%) | 8.4 ± 6.1 (1-32) |
|  |  |  |  |  | Rheumatoid arthritis | 48 (27.6%) | 10.1 ± 7.9 (1-29) |
|  |  |  |  |  | Celiac disease | 45 (25.9%) | 6.3 ± 5.0 (1-19) |
|  |  |  |  |  | Systemic lupus erythematosus | 12 (6.9%) | 9.1 ± 8.3 (1-27) |
|  |  |  |  |  | Chronic atrophic gastritis | 6 (3.4%) | 16.2 ± 16.2 (1-44) |
|  |  |  |  |  | Vitiligo | 5 (2.9%) | 10.8 ± 6.8 (3-18) |
|  |  |  |  |  | Autoimmune hepatitis | 3 (1.7%) | 8.0 ± 6.6 (1-14) |
|  |  |  |  |  | Psoriasis | 3 (1.7%) | 9.0 ± 3.5 (7-13) |
|  |  |  |  |  | Premature ovarian failure | 2 (1.2%) | 1.0 ± 0.0 (1-1) |
|  |  |  |  |  | Sjogren’s syndrome | 2 (1.2%) | 7.5 ± 0.7 (7-8) |
|  |  |  |  |  | Mixed connective tissue disease | 2 (1.2%) | 5.0 ± 4.2 (2-8) |
|  |  |  |  |  | Inflammatory bowel disease | 1 (0.6%) | 7 |
|  |  |  |  |  | Chronic hypoparathyroidism | 1 (0.6%) | 11 |
|  |  |  |  |  | Alopecia areata | 1 (0.6%) | 1 |
|  |  |  |  |  | Autoimmune anemia | 1 (0.6%) | 1 |
| Type I diabetes mellitus and autoimmune thyroiditis | 87 (8.3%) | 39/48 | 28.9 ± 14.7 | 0 | Celiac disease | 1 (1.1%) | 13 |
| Autoimmune thyroiditis and celiac disease | 80 (7.6%) | 17/63 | 40.3 ± 14.0 | 0 | Rheumatoid arthritis | 2 (2.5%) | 2.5 ± 0.7 (2-3) |
|  |  |  |  |  | Psoriasis | 1 (1.3%) | 7 |
|  |  |  |  |  | Seronegative arthritis | 1 (1.3%) | 8 |
|  |  |  |  |  | Chronic atrophic gastritis | 1 (1.3%) | 1 |
| Celiac disease | 78 (7.5%) | 16/62 | 34.0 ± 13.8 | 5.3 ± 6.1 (1-29) | Autoimmune thyroiditis | 72 (92.3%) | 5.3 ± 6.1 (1-27) |
|  |  |  |  |  | Type I diabetes mellitus | 9 (11.5%) | 9.4 ± 6.3 (2-20) |
|  |  |  |  |  | Graves’ disease | 6 (7.7%) | 14.0 ± 12.5 (1-29) |
|  |  |  |  |  | Premature ovarian failure | 2 (3.2%) | 14.5 ± 3.5 (12-17) |
|  |  |  |  |  | Chronic atrophic gastritis | 1 (1.3%) | 13 |
|  |  |  |  |  | Rheumatoid arthritis | 1 (1.3%) | 23 |
|  |  |  |  |  | Sjogren’s syndrome | 1 (1.3%) | 12 |
|  |  |  |  |  | Autoimmune hepatitis | 1 (1.3%) | 10 |
|  |  |  |  |  | Vitiligo | 1 (1.3%) | 9 |
|  |  |  |  |  | Psoriasis | 1 (1.3%) | 18 |
| Rheumatoid arthritis | 72 (6.9%) | 9/63 | 55.2 ± 15.4 | 9.7 ± 8.7 (1-51) | Autoimmune thyroiditis | 67 (93.1%) | 9.9 ± 8.8 (1-51) |
|  |  |  |  |  | Graves’ disease | 5 (6.9%) | 7.4 ± 7.8 (1-21) |
|  |  |  |  |  | Seronegative arthritis | 2 (2.8%) | 9.5 ± 9.2 (3-16) |
|  |  |  |  |  | Celiac disease | 1 (1.4%) | 12 |
|  |  |  |  |  | Vitiligo | 1 (1.4%) | 17 |
|  |  |  |  |  | Autoimmune urticaria | 1 (1.4%) | 1 |
| Vitiligo | 38 (3.6%) | 10/28 | 41.2 ± 16.3 | 16.3 ± 14.3 (1-66) | Autoimmune thyroiditis | 31 (81.6%) | 15.4 ± 12.3 (1-50) |
|  |  |  |  |  | Graves’ disease | 7 (18.4%) | 20.2 ± 23.6 (4-66) |
|  |  |  |  |  | Chronic atrophic gastritis | 4 (10.5%) | 26.3 ± 8.2 (14-31) |
|  |  |  |  |  | Type I diabetes mellitus | 2 (5.3%) | 9.5 ± 0.7 (9-10) |
|  |  |  |  |  | Celiac disease | 2 (5.3%) | 11.5 ± 7.8 (6-17) |
|  |  |  |  |  | Inflammatory bowel disease | 1 (2.6%) | 6 |
|  |  |  |  |  | Sjogren’s syndrome | 1 (2.6%) | 15 |
|  |  |  |  |  | Premature ovarian failure | 1 (2.6%) | 11 |
|  |  |  |  |  | Seronegative arthritis | 1 (2.6%) | 5 |
|  |  |  |  |  | Pemphigoid | 1 (2.6%) | 2 |
| Graves’ disease | 37 (3.5%) | 2/35 | 44.2 ± 14.7 | 11.2 ± 11.8 (1-62) | Celiac disease | 16 (43.2%) | 9.0 ± 8.3 (1-30) |
|  |  |  |  |  | Rheumatoid arthritis | 7 (18.9%) | 14.9 ± 21.5 (1-62) |
|  |  |  |  |  | Type I diabetes mellitus | 5 (13.5%) | 10.0 ± 9.8 (1-26) |
|  |  |  |  |  | Vitiligo | 3 (8.1%) | 12.7 ± 9.3 (2-19) |
|  |  |  |  |  | Chronic atrophic gastritis | 3 (8.1%) | 13.3 ± 10.7 (1-20) |
|  |  |  |  |  | Autoimmune thyroiditis | 2 (5.4%) | 8.0 ± 5.7 (4-12) |
|  |  |  |  |  | Systemic lupus erythematosus | 2 (5.4%) | 13.0 ± 17.0 (1-25) |
|  |  |  |  |  | Sjogren’s syndrome | 2 (5.4%) | 4.0 ± 2.8 (2-6) |
|  |  |  |  |  | Mixed connective tissue disease | 1 (2.7%) | 16 |
|  |  |  |  |  | Antiphospholipid syndrome | 1 (2.7%) | 8 |
|  |  |  |  |  | Autoimmune hepatitis | 1 (2.7%) | 23 |
|  |  |  |  |  | Psoriasis | 1 (2.7%) | 16 |
|  |  |  |  |  | Seronegative arthritis | 1 (2.7%) | 20 |
| Systemic lupus erythematosus | 16 (1.5%) | 1/15 | 42.2 ± 12.9 | 9.7 ± 7.9 (1-30) | Autoimmune thyroiditis | 13 (81.3%) | 8.9 ± 7.8 (1-30) |
|  |  |  |  |  | Graves’ disease | 3 (18.7%) | 15.0 ± 8.7 (10-25) |
|  |  |  |  |  | Type I diabetes mellitus | 2 (12.5%) | 8.0 ± 2.8 (6-10) |
|  |  |  |  |  | Chronic atrophic gastritis | 1 (6.3%) | 11 |
|  |  |  |  |  | Seronegative arthritis | 1 (6.3%) | 19 |
| Autoimmune thyroiditis and rheumatoid arthritis | 15 (1.4%) | 0/15 | 55.1 ± 17.8 | 0 | NA | NA | NA |
| Chronic atrophic gastritis | 6 (0.6%) | 2/4 | 51.8 ± 11.3 | 4.2 ± 4.3 (1-12) | Autoimmune thyroiditis | 5 (83.3%) | 3.8 ± 4.7 (1-12) |
|  |  |  |  |  | Graves’ disease | 1 (16.7%) | 6 |
|  |  |  |  |  | Rheumatoid arthritis | 1 (16.7%) | 1 |
|  |  |  |  |  | Premature ovarian failure | 1 (25%) | 13 |
| Multiple sclerosis | 6 (0.6%) | 0/6 | 46.8 ± 13.7 | 13.2 ± 14.0 (2-40) | Autoimmune thyroiditis | 6 (100%) | 13.2 ± 14.0 (2-40) |
|  |  |  |  |  | Graves’ disease | 1 (16.7%) | 47 |
| Type I diabetes mellitus and Graves’ disease | 6 (0.6%) | 2/4 | 35.0 ± 10.1 | 0 | Rheumatoid arthritis | 1 (16.7%) | 20 |
| Sjogren’s syndrome and rheumatoid arthritis | 6 (0.6%) | 0/6 | 45.3 ± 14.0 | 11.0 ± 7.4 (1-17) | Autoimmune thyroiditis | 6 (100%) | 11.0 ± 7.4 (1-17) |
|  |  |  |  |  | Psoriasis | 1 (16.7%) | 23 |
| Alopecia areata | 5 (0.5%) | 2/3 | 44.2 ± 15.2 | 8.4 ± 9.9 (3-26) | Autoimmune thyroiditis | 5 (100%) | 8.4 ± 9.9 (3-26) |
|  |  |  |  |  | Celiac disease | 2 (40.0%) | 3.5 ± 3.5 (1-6) |
|  |  |  |  |  | Rheumatoid arthritis | 2 (40.0%) | 6.5 ± 0.7 (6-7) |
|  |  |  |  |  | Inflammatory bowel disease | 1 (20.0%) | 40 |
|  |  |  |  |  | Ankylosing spondylitis | 1 (20.0%) | 10 |
| Psoriasis | 5 (0.5%) | 0/5 | 43.6 ± 11.4 | 14.2 ± 17.3 (2-39) | Autoimmune thyroiditis | 4 (80.0%) | 11.3 ± 18.5 (2-39) |
|  |  |  |  |  | Rheumatoid arthritis | 2 (40.0%) | 28.5 ± 12.0 (20-37) |
|  |  |  |  |  | Graves’ disease | 1 (20.0%) | 26 |
|  |  |  |  |  | Seronegative arthritis | 1 (20.0%) | 9 |
| Autoimmune thyroiditis and chronic atrophic gastritis | 5 (0.5%) | 2/3 | 53.2 ± 9.2 | 0 | NA | NA | NA |
| Autoimmune thyroiditis and systemic lupus erythematosus | 5 (0.5%) | 0/5 | 40.0 ± 14.3 | 0 | Immune thrombocytopenia | 1 (20.0%) | 7 |
| Autoimmune thyroiditis and vitiligo | 5 (0.5%) | 3/2 | 37.4 ± 11.5 | 0 | Chronic atrophic gastritis | 1 (20.0%) | 1 |
| Premature ovarian failure | 4 (0.5%) | 0/4 | 53.5 ± 12.4 | 19.5 ± 17.5 (2-43) | Autoimmune thyroiditis | 3 (75.0%) | 11.7 ± 19.5 (2-21) |
|  |  |  |  |  | Graves’ disease | 1 (25.0%) | 43 |
|  |  |  |  |  | Type I diabetes mellitus | 1 (25.0%) | 26 |
|  |  |  |  |  | Chronic atrophic gastritis | 1 (25.0%) | 2 |
| Sjogren’s syndrome | 3 (0.3%) | 0/3 | 42.3 ± 17.6 | 3.0 ± 1.7 (2-5) | Autoimmune thyroiditis | 3 (100%) | 3.0 ± 1.7 (2-5) |
|  |  |  |  |  | Chronic atrophic gastritis | 1 (33.3%) | 2 |
| Mixed connective tissue disease | 3 (0.3%) | 1/2 | 54.0 ± 12.0 | 4.0 ± 2.0 (2-6) | Autoimmune thyroiditis | 3 (100%) | 4.0 ± 2.0 (2-6) |
|  |  |  |  |  | Type I diabetes mellitus | 1 (33.3%) | 5 |
|  |  |  |  |  | Chronic atrophic gastritis | 1 (33.3%) | 5 |
| Primary biliary cirrhosis | 3 (0.3%) | 1/2 | 60.3 ± 5.1 | 21.7 ± 11.4 (9-31) | Autoimmune thyroiditis | 3 (100%) | 21.7 ± 11.4 (9-31) |
|  |  |  |  |  | Sjogren’s syndrome | 1 (33.3%) | 16 |
|  |  |  |  |  | Autoimmune hypoparathyroidism | 1 (33.3%) | 9 |
| Autoimmune urticaria | 3 (0.3%) | 0/3 | 26.7 ± 6.8 | 12.3 ± 12.1 (1-25) | Autoimmune thyroiditis | 3 (100%) | 12.3 ± 12.1 (1-25) |
|  |  |  |  |  | Alopecia areata | 1 (33.3%) | 11 |
|  |  |  |  |  | Systemic lupus erythematosus | 1 (33.3%) | 17 |
| Type I diabetes mellitus and celiac disease | 3 (0.3%) | 1/2 | 23.7 ± 14.6 | 0 | Autoimmune thyroiditis | 3 (100%) | 4.7 ± 3.8 (2-9) |
| Autoimmune thyroiditis and alopecia areata | 3 (0.3%) | 1/2 | 28.0 ± 14.9 | 0 | Chronic atrophic gastritis | 1 (33.3%) | 11 |
| Immune thrombocytopenia | 2 (0.2%) | 0/2 | 20.0 ± 1.4 | 3.5 ± 2.1 (2-5) | Autoimmune thyroiditis | 2 (100%) | 3.5 ± 2.1 (2-5) |
|  |  |  |  |  | Systemic lupus erythematosus | 1 (50.0%) | 12 |
| Type I diabetes mellitus, autoimmune thyroiditis, and celiac disease | 2 (0.2%) | 1/1 | 28.5 ± 13.4 | 0 | NA | NA | NA |
| Graves’ disease and celiac disease | 2 (0.2%) | 0/2 | 31.5 ± 3.5 | 0 | NA | NA | NA |
| Autoimmune thyroiditis and premature ovarian failure | 2 (0.2%) | 0/2 | 39.5 ± 6.4 | 0 | NA | NA | NA |
| Inflammatory bowel disease | 1 (0.1%) | 0/1 | 44 | 11 | Autoimmune thyroiditis | 1 (100%) | 11 |
| Myasthenia gravis | 1 (0.1%) | 1/0 | 28 | 12 | Autoimmune thyroiditis | 1 (100%) | 12 |
| Autoimmune hepatitis | 1 (0.1%) | 1/0 | 64 | 6 | Autoimmune thyroiditis | 1 (100%) | 6 |
| Ankylosing spondylitis | 1 (0.1%) | 0/1 | 54 | 5 | Autoimmune thyroiditis | 1 (100%) | 5 |
| Type I diabetes mellitus, autoimmune thyroiditis, and scleroderma | 1 (0.1%) | 0/1 | 45 | 0 | NA | NA | NA |
| Autoimmune thyroiditis, rheumatoid arthritis, and vitiligo | 1 (0.1%) | 1/0 | 57 | 0 | NA | NA | NA |
| Autoimmune thyroiditis, celiac disease, and chronic atrophic gastritis | 1 (0.1%) | 0/1 | 61 | 0 | NA | NA | NA |
| Celiac disease and chronic atrophic gastritis | 1 (0.1%) | 0/1 | 54 | 1 | Autoimmune thyroiditis | 1 (100%) | 1 |
| Rheumatoid arthritis and pernicious anemia | 1 (0.1%) | 0/1 | 26 | 1 | Autoimmune thyroiditis | 1 (100%) | 1 |
| Rheumatoid arthritis and psoriasis | 1 (0.1%) | 0/1 | 51 | 5 | Autoimmune thyroiditis | 1 (100%) | 5 |
| Immune thrombocytopenia and systemic lupus erythematosus | 1 (0.1%) | 0/1 | 16 | 30 | Autoimmune thyroiditis | 1 (100%) | 30 |
| Autoimmune thyroiditis and antiphospholipid syndrome | 1 (0.1%) | 0/1 | 42 | 0 | NA | NA | NA |
| Autoimmune thyroiditis, celiac disease and autoimmune urticaria | 1 (0.1%) | 0/1 | 27 | 0 | NA | NA | NA |
| Graves’ disease, vitiligo, and myasthenia gravis | 1 (0.1%) | 0/1 | 24 | 0 | NA | NA | NA |
| Autoimmune thyroiditis, systemic lupus erythematosus, and vitiligo | 1 (0.1%) | 0/1 | 59 | 0 | NA | NA | NA |
| Graves’ disease and vitiligo | 1 (0.1%) | 1/0 | 46 | 0 | NA | NA | NA |

NA: not applicable
