# Supplementary material for: Evaluation of a large set of patients with Autoimmune Polyglandular Syndrome from a single reference centre in context of different classifications
Source: J Endocrinol Invest. 2023 Sep 26;47(4):857–64. doi: 10.1007/s40618-023-02200-6 (PMC10965644; doi:10.1007/s40618-023-02200-6)
Supplement: Supplementary file 4 — Supplementary file4 (DOCX 18 KB) [file 40618_2023_2200_MOESM4_ESM.docx]

**Supplementary** **file 4.** Demographic and clinical characteristics of patients diagnosed with APS-4.

| **First disease** | **Number of patients (%)** | **Sex M/F** | **Mean age of diagnosis** | **Latency from APS diagnosis and range (years)** | **Subsequent diseases** | **Number of patients (%)** | **Latency from the first disease and range (years)** |
| --- | --- | --- | --- | --- | --- | --- | --- |
| Type I diabetes mellitus | 72 (75.0%) | 32/40 | 22.0 ± 15.1 | 13.6 ± 11.1 (1-46) | Celiac disease | 35 (48.6%) | 7.5 ± 7.3  (1 – 28) |
|  |  |  |  |  | Rheumatoid arthritis | 9 (12.5%) | 19.8 ± 11.6  (3 – 38) |
|  |  |  |  |  | Systemic lupus erythematosus | 6 (8.3%) | 14.2 ± 13.0  (2 – 37) |
|  |  |  |  |  | Vitiligo | 6 (8.3%) | 18.8 ± 13.0  (2 – 40) |
|  |  |  |  |  | Seronegative arthritis | 5 (6.9%) | 23.4 ± 11.0  (12 – 36) |
|  |  |  |  |  | Inflammatory bowel disease | 4 (5.6%) | 25.0 ± 8.0  (16 – 34) |
|  |  |  |  |  | Multiple sclerosis | 4 (5.6%) | 23.0 ± 6.6  (14 – 30) |
|  |  |  |  |  | Atrophic gastritis | 3 (4.2%) | 9.3 ± 6.1  (4 – 16) |
|  |  |  |  |  | Psoriasis | 3 (4.2%) | 27.0 ± 17.3  (12 – 46) |
|  |  |  |  |  | Mixed connective tissue disease | 2 (2.8%) | 25.0 ± 5.7  (21 – 29) |
|  |  |  |  |  | Vasculitis | 1 (1.4%) | 8 |
|  |  |  |  |  | Immune thrombocytopenia | 1 (1.4%) | 3 |
| Type I diabetes mellitus and celiac disease | 13 (86.7%) | 8/5 | 23.5 ± 14.3 | 0 | NA | NA | NA |
| Celiac disease | 9 (9.4%) | 4/5 | 24.0 ± 19.8 | 13.0 ± 10.1  (1 – 29) | Type I diabetes mellitus | 9 (100%) | 13.0 ± 10.1  (1 – 29) |
| Vitiligo | 4 (4.2%) | 2/2 | 19.5 ± 15.2 | 10.0 ± 7.6 (2 – 20) | Type I diabetes mellitus | 3 (75%) | 9.7 ± 9.3  (2 – 20) |
|  |  |  |  |  | Premature ovarian failure | 1 (25%) | 11 |
| Psoriasis | 3 (3.1%) | 2/1 | 18.3 ± 6.5 | 10.3 ± 5.1  (6 – 16) | Type I diabetes mellitus | 3 (100%) | 10.3 ± 5.1  (6 – 16) |
| Type I diabetes mellitus and multiple sclerosis | 2 (13.3%) | 0/2 | 23.5 ± 4.9 | 0 | NA | NA | NA |
| Inflammatory bowel disease | 2 (2.1%) | 1/1 | 34.0 ± 26.9 | 19.5 ± 10.7  (19 – 20) | Type I diabetes mellitus | 2 (100%) | 19.5 ± 10.7  (19 – 20) |
| Rheumatoid arthritis | 2 (2.1%) | 0/2 | 28.5 ± 3.5 | 19.5 ± 3.5 (17 – 22) | Type I diabetes mellitus | 1 (50%) | 22 |
|  |  |  |  |  | Premature ovarian failure | 1 (50%) | 17 |
| Premature ovarian failure | 1 (1.0%) | 0/1 | 30 | 7 | Rheumatoid arthritis | 1 (100%) | 7 |
| Seronegative arthritis | 1 (1.0%) | 0/1 | 42 | 2 | Type I diabetes mellitus | 1 (100%) | 2 |
| Ankylosing spondylitis | 1 (1.0%) | 1/0 | 42 | 10 | Type I diabetes mellitus | 1 (100%) | 10 |
| Primary biliary cirrhosis | 1 (1.0%) | 1/0 | 41 | 20 | Vitiligo and Type I diabetes mellitus | 1 (100%) | 20 |

NA: not applicable
